# The statistical analysis plan for the unification of treatments and interventions for tinnitus patients randomized clinical trial (UNITI-RCT)

**DOI:** 10.1186/s13063-023-07303-2

**Published:** 2023-07-24

**Authors:** Jorge Piano Simoes, Stefan Schoisswohl, Winfried Schlee, Laura Basso, Alberto Bernal-Robledano, Benjamin Boecking, Rilana Cima, Sam Denys, Milena Engelke, Alba Escalera-Balsera, Alvaro Gallego-Martinez, Silvano Gallus, Dimitris Kikidis, Jose A. López-Escámez, Steven C. Marcrum, Nikolaos Markatos, Juan Martin-Lagos, Marta Martinez-Martinez, Birgit Mazurek, Evgenia Vassou, Carlotta Micaela Jarach, Nicolas Mueller-Locatelli, Patrick Neff, Uli Niemann, Hafez Kader Omar, Clara Puga, Miro Schleicher, Vishnu Unnikrishnan, Patricia Perez-Carpena, Rüdiger Pryss, Paula Robles-Bolivar, Matthias Rose, Martin Schecklmann, Tabea Schiele, Johannes Schobel, Myra Spiliopoulou, Sabine Stark, Carsten Vogel, Nina Wunder, Zoi Zachou, Berthold Langguth

**Affiliations:** 1 Department of Psychiatry and Psychotherapy, University of Regensburg, Regensburg, Germany; 2 Department of Psychology, Health and Technology, University of Twente, Enschede, The Netherlands; 3 Department of Psychology, Universität der Bundeswehr München, Neubiberg, Germany; 4 Institute for Information and Process Management, Eastern Switzerland University of Applied Sciences, St. Gallen, Switzerland; 5 Department of Otolaryngology, Instituto de Investigación Biosanitaria ibs.Granada, Hospital Universitario Virgen de Las Nieves, Universidad de Granada, 18014 Granada, Spain; 6 Tinnitus Center, Charité – Universitätsmedizin Berlin, Corporate Member of Freie Universität and Humboldt-Universität zu Berlin, Charitéplatz 1, 10117 Berlin, Germany; 7 Health Psychology, Faculty of Psychology and Educational Sciences, KU Leuven University, Leuven, Belgium; 8 Tinnitus Center of Expertise, Centre of Expertise in Rehabilitation and Audiology, Adelante, Hoensbroek, The Netherlands; 9 Experimental Health Psychology, Faculty of Psychology and Neurosciences, Maastricht University, Maastricht, The Netherlands; 10 Research group Experimental Otorhinolaryngology (ExpORL), Department of Neurosciences, KU Leuven University, Leuven, Belgium; 11 Department of Otorhinolaryngology, Head and Neck surgery, University Hospitals of Leuven, Leuven, Belgium; 12 Otology & Neurotology Group CTS 495, Department of Genomic Medicine, GENYO, Center for Genomics and Oncological Research: Pfizer/University of Granada/Andalusian Regional Government, Granada, Spain; 13 Istituto Di Ricerche Farmacologiche Mario Negri IRCCS, Milan, Italy; 14 Department of Otorhinolaryngology, Head and Neck Surgery, National and Kapodistrian University of Athens, Medical School, Athens, Greece; 15 Department of Surgery, Division of Otolaryngology, Faculty of Medicine, University of Granada, Granada, Spain; 16 Meniere’s Disease Neuroscience Research Program, Faculty of Medicine & Health, School of Medical Sciences, The Kolling Institute, University of Sydney, Sydney, New South Wales Australia; 17 Department of Otolaryngology, University Hospital Regensburg, Regensburg, Germany; 18 Department of Otolaryngology, Instituto de Investigacion Biosanitaria Granada, ibs.GRANADA, Hospital Universitario San Cecilio, Granada, Spain; 19 Department of Otorhinolaryngology, Head & Neck Surgery, University Hospital Zurich, University of Zurich, Zurich, Switzerland; 20 Neuro-X Institute, École Polytechnique Fédérale de Lausanne (EPFL), Geneva, Switzerland; 21 Centre for Cognitive Neuroscience, University of Salzburg, Salzburg, Austria; 22 Knowledge Management and Discovery Lab (KMD), Faculty of Computer Science, Otto Von Guericke University Magdeburg, Magdeburg, Germany; 23 Institute of Clinical Epidemiology and Biometry, University of Würzburg, Würzburg, Germany; 24 Department of Psychosomatic Medicine, Center for Internal Medicine and Dermatology, Charité – Universitätsmedizin Berlin, Corporate Member of Freie Universität and Humboldt-Universität zu Berlin, Berlin, Germany; 25 Institute DigiHealth, University of Applied Sciences, Neu-Ulm, Germany

## Abstract

**Background:**

Tinnitus is a leading cause of disease burden globally. Several therapeutic strategies are recommended in guidelines for the reduction of tinnitus distress; however, little is known about the potentially increased effectiveness of a combination of treatments and personalized treatments for each tinnitus patient.

**Methods:**

Within the Unification of Treatments and Interventions for Tinnitus Patients project, a multicenter, randomized clinical trial is conducted with the aim to compare the effectiveness of single treatments and combined treatments on tinnitus distress (UNITI-RCT). Five different tinnitus centers across Europe aim to treat chronic tinnitus patients with either cognitive behavioral therapy, sound therapy, structured counseling, or hearing aids alone, or with a combination of two of these treatments, resulting in four treatment arms with single treatment and six treatment arms with combinational treatment. This statistical analysis plan describes the statistical methods to be deployed in the UNITI-RCT.

**Discussion:**

The UNITI-RCT trial will provide important evidence about whether a combination of treatments is superior to a single treatment alone in the management of chronic tinnitus patients. This pre-specified statistical analysis plan details the methodology for the analysis of the UNITI trial results.

**Trial registration:**

ClinicalTrials.gov NCT04663828. The trial is ongoing. Date of registration: December 11, 2020. All patients that finished their treatment before 19 December 2022 are included in the main RCT analysis.

## Background

Tinnitus is a common condition associated with a high global disease burden. Currently, there is no universal treatment or cure for tinnitus [1]. Different therapeutic strategies are recommended to reduce the burden of tinnitus; however, little is known about the potentially greater effectiveness of a combination of treatments compared to single treatments. Moreover, treatment studies in tinnitus research often suffer from methodological shortcomings. High-quality multi-center randomized clinical trials (RCTs) could help to achieve methodologically more robust results with greater external validity. Within the multidisciplinary EU-funded project “Unification of Treatment and Interventions for Tinnitus Patients” (UNITI [1]), a multicenter randomized clinical trial (UNITI-RCT) is conducted with the aim to compare the effectiveness of single or combinational treatment interventions for tinnitus. In detail, there are 10 different treatment arms: four single treatments (cognitive behavioral therapy (CBT), sound therapy (ST), structured counseling (SC), or hearing aids (HA)) and all six possible two-treatment combinations of the respective single treatments (CBT and HA, CBT and SC, CBT and ST, HA and SC, HA and ST, SC and ST).

A study protocol for UNITI-RCT has previously been published [1], and the trial has been registered at ClinicalTrials.gov (NCT04663828). The study protocol states that the main goal of UNITI-RCT is to “[…] overcome the shortcomings of previous studies, but also pave the way for personalized medicine approaches in tinnitus. For this purpose, a multi-center parallel-arm superiority RCT, implemented and harmonized among five clinical sites across the EU, combining and investigating selected existing therapies evaluated in the European guidelines for tinnitus [2], is conducted.”

The UNITI-RCT is executed in five clinical centers across the EU. All patients that finished their treatment before 19 December 2022 are included in the main RCT analysis. The already published study protocol delineates the rationale and methods of the study, its population plus the respective inclusion and exclusion criteria, the description of outcome measures, collected covariates, and the used interventions. As a follow-up to the study’s protocol, this statistical analysis plan (SAP) aims to further describe the statistical techniques in more detail used to address the primary objectives of the RCT. To increase the transparency of data analysis, this plan will be made public before database closure (UNITI website: https://uniti.tinnitusresearch.net/index.php/169-start-of-statistical-analysis-of-rct/ preprint: https://www.researchsquare.com/article/rs-2123725/v1) and thus prior to the beginning of data analysis of the main objectives of the UNITI-RCT.

## Methods/design

### Study objectives

As stated in the study protocol [1], the objectives of the UNITI-RCT are to examine whether:

- (1) Combination therapy is more effective than a single therapy for the treatment of chronic tinnitus;
- (2) The effectiveness of the ten investigated interventions differs from each other;
- (3) For the four treatment types (SC, ST, HA, CBT) the combination with another treatment is superior to the treatment alone;
- (4) A certain type of intervention either alone or in combination is superior to other treatments;
- (5) A combination of treatments targeting both the auditory system and the central nervous system are superior to treatments targeting only either the ear or the brain;
- (6) The development of a Decision Support System (DSS), where machine learning will be used to deliver personalized suggestions for interventions aiming to maximize its effectiveness.

This SAP describes how objectives 1–5 will be evaluated; see Table 1. All these objectives are testing for the superiority of one or several treatment types over the others. The first objective, which focuses on comparing the effects of single and combinatorial treatments in general and independent from the specific intervention, will be considered the main objective to be addressed by UNITI-RCT. The development of the DSS (objective 6) will be described elsewhere.

**Table 1:** Overview of the planned analyses to address the objectives of the UNITI-RCT

**Objective**	**Description of comparison**	**Contrasted groups**	**Primary outcome**	**Secondary outcome**					
1	**Single versus combined**	(CBT, ST, SC, HA) versus (CBT + HA, CBT + ST, CBT + SC, ST + HA, ST + SC, SC + HA)	THI	CGI-I	TFI	Mini TQ	NRS	WHO-QoL Bref	PHQ-9
2	**All ten treatment arms**	CBT, ST, SC, HA, CBT + HA, CBT + ST, CBT + SC, ST + HA, ST + SC, SC + HA versus each other							
3	**SC single versus combined**	SC versus (SC + CBT, SC + ST, SC + HA)							
	**ST single versus combined**	ST versus (ST + SC, ST + CBT, ST + HA)							
	**HA single versus combined**^**a**^	HA versus (HA + SC, HA + CBT, HA + ST)							
	**CBT single versus combined**	CBT versus (CBT + SC, CBT + ST, CBT + HA)							
4	**SC versus no SC**	(SC, CBT + SC, ST + SC, SC + HA) versus (CBT, ST, HA, CBT + HA, CBT + ST, ST + HA)							
	**ST versus no ST**	(ST, CBT + ST, ST + HA, ST + SC) versus (CBT, SC, HA, CBT + HA, CBT + SC, SC + HA)							
	**HA versus no HA**^**a**^	(HA, CBT + HA, ST + HA, SC + HA) versus (CBT, ST, SC, CBT + ST, CBT + SC, ST + SC)							
	**CBT versus no CBT**	(CBT, CBT + HA, CBT + ST, CBT + SC) versus (ST, SC, HA, ST + HA, ST + SC, SC + HA)							
5	**Combination of brain and ear targeting treatments versus ear or brain targeting treatments**	(CBT + HA, CBT + ST, ST + SC, SC + HA) versus (CBT, SC, CBT + SC) versus (HA, ST, HA + ST)							

*CBT* cognitive behavior therapy, *ST* sound therapy, *SC* structured counseling, *HA* hearing aids

^a^Only patients from the strata with HA indication are included in this analysis

### Patient population

Each center aims to enroll 100 patients for the RCT, for a total number of 500 patients with chronic subjective tinnitus (i.e., lasting for at least 6 months). At all sites, potential candidates are recruited via media advertising (according to local regulations) as well as on an individual basis at the clinical sites through, e.g., information sheets, word of mouth, or conversations with medical staff.

### Inclusion and exclusion criteria

Tables 2 and 3 summarize the inclusion and exclusion criteria for UNITI-RCT.

**Table 2:** Inclusion criteria of UNITI-RCT as specified in the study protocol [1]

**Inclusion criteria**
Tinnitus as the primary complaint
Tinnitus lasting at least 6 months
Age 18–80 years
A score ≥ 18 in the Tinnitus Handicap Inventory at Screening
A score greater than 22 at the Montreal Cognitive Assessment (MoCa)
Ability and willingness to use the UNITI mobile applications [3] on their smartphones
Openness to using a hearing aid (if allocation to the hearing aid stratum)
Ability to understand and consent to the research (hearing ability, intellectual capacity)
Ability to participate in all relevant visits (no plans for, e.g., long-term holidays or pregnancy^a^)
Negative pregnancy test at screening (only at the clinical site in Granada due to specific standards of the local ethics committee)
Existing drug therapy with psychoactive substances (e.g., antidepressants, anticonvulsants) must be stable for at least 30 days at the beginning of the therapeutic intervention. The drug therapy should remain constant during the duration of the study. Necessary changes do not constitute an exclusion criterion per se but need to be recorded

^a^Due to specific standards of the local ethics committee at the clinical site in Granada, Spain, with respect to the conduction of RCTs, all female participants will be tested with regard to an existing pregnancy

**Table 3:** Exclusion criteria of UNITI-RCT as specified in the study protocol [1]

**Exclusion criteria**
Objective tinnitus or heartbeat- synchronous tinnitus as primary complaint
Otosclerosis/acoustic neuroma or other relevant ear disorders with fluctuation hearing
Present acute infections (acute otitis media, otitis externa, acute sinusitis)
Meniere's disease or similar syndromes with the exception of vestibular migraine
Serious internal, neurological or psychiatric conditions
Epilepsy or other central nervous system disorders (brain tumor, encephalitis)
Clinically relevant drug, medication or alcohol abuse up to 12 weeks before study start
Severe hearing loss as defined by the inability to communicate properly in the course of the study
At least one deaf ear
Missing written informed consent
Start of any other tinnitus-related treatments, especially hearing aids, structured counseling, sound therapy (with special devices; expecting long-term effects) or cognitive behavioral therapy in the last 3 months before the start of the study^a^

^a^If a HA has already been worn 3 months before screening, eligible candidates are allowed to participate, but are automatically assigned to the group with no HA indication

### Outcomes

The Tinnitus Handicap Inventory (THI) will be used as a primary outcome measure (see Table 1). In addition to the THI, secondary outcome measures are the Tinnitus Functional Index (TFI [4]), the short version of the Tinnitus Questionnaire (mini-TQ [5]), Tinnitus Numeric Rating Scales (NRS [6]), World Health Organization – Quality of Life abbreviated (WHOQoL-Bref; https://www.who.int/healthinfo/survey/WHOQOL_BREF.pdf?ua=1), Clinical Global Impression Scale—Improvement (CGI-I [7]), and Patient Health Questionnaire for Depression (PHQ-9 [8]).

Additional measures which are not defined as primary or secondary outcomes but may be used for sample description and additional analyses include: European School of Interdisciplinary Tinnitus Research Screening Questionnaire (ESIT-SQ [9]), Tinnitus Sample Case History Questionnaire (TSCHQ [10]), Questionnaire on Hypersensitivity to Sound (GUF) [11], Big Five Inventory 2 (BFI-2 [12]), Montreal Cognitive Assessment (MoCA, also used as inclusion criteria, see Table 2 [13]), a short version of the Social Isolation Electronic Survey (Mini-SOISES [14]), Attitudes Towards Amplification Questionnaire (ATAQ) which consists of a subset of questions from the Attitudes towards Loss of Hearing Questionnaire (ALHQ [15]), Fear of Tinnitus Questionnaire (FTQ [16]), and audiometric and tinnitometric measurements (e.g., tinnitus loudness and frequency, maskability with minimum masking levels, and residual inhibition).

### Variables assessment

An overview of all study assessments and the time points when they were collected is presented in Table 4. The visit window for each study visit was ± 7 days. In addition to the outcome and other clinical measures described above, the assessment included voluntary blood sampling, auditory brainstem response (ABR) and auditory middle-latency responses (AMLR), and recording of concomitant treatment/medication. The collected ABR and AMLR data and blood samples will be addressed in additional analyses to the one described here. Safety measures are otological examination, audiometry, comorbidities, and adverse effects.

**Table 4:** Overview of assessments for the UNITI-RCT

	**Pre-screening**	**Screening**	**Baseline**	**Treatment start**	**Interim visit**	**Final visit = end of treatment**	**Follow-up**	**Additional follow-up**
ICF	**A**^**a**^	**A**						
Eligibility criteria	**A**	**A**	**A**					
ESIT-SQ			**A**					
TSCHQ			**B**					
Mini TQ	**A**	**A**	**A**		**A**	**A**	**A**	**B**
Tinnitus numeric rating scales		**A**	**A**		**A**	**A**	**A**	**B**
TFI		**A**	**A**		**A**	**A**	**A**	**B**
THI	**A**	**A**	**A**		**A**	**A**	**A**	**B**
WhoQol-BREF		**A**	**A**		**A**	**A**	**A**	**B**
BFI-2			**A**					
CGI-I					**A**	**A**	**A**	**B**
GUF		**B**	**B**		**B**	**B**	**B**	**B**
PHQ-D	**A**	**A**	**A**		**A**	**A**	**A**	**B**
Mini-SOISES			**A**		**A**	**A**	**A**	**B**
ATAQ			**B**^**b**^			**B**^**b**^		
FTQ			**B**		**B**	**B**	**B**	**B**
MoCA		**A**						
Randomization			**A**					
Blood sampling			**B**^**c**^					
Otological examination		**A**				**A**	**B**	**B**
Audiometry		**A**				**A**	**B**	**B**
Loudness match		**A**				**A**	**B**	**B**
Pitch match		**A**				**A**	**B**	**B**
Maskability		**A**				**A**	**B**	**B**
Residual inhibition		**A**				**B**	**B**	**B**
ABR			**A**				**B**	**B**
AMLR			**A**				**B**	**B**
Treatment				**A**	**A**	**A**		
Comorbidities		**A**	**A**	**A**	**A**	**A**	**A**	**B**
Concomitant medication/ treatment		**A**	**A**	**A**	**A**	**A**	**A**	**B**
Adverse events					**A**	**A**	**A**	**B**

Table reproduced from [1] (CC BY 4.0). Interim visit: week 6; final visit: week 12; follow-up: week 36; additional follow-up: week 48. *A* mandatory, *B* voluntary, *ICF* Informed Consent Form, *ESIT-SQ* European School of Interdisciplinary Tinnitus Research Screening Questionnaire, *TSCHQ* Tinnitus Sample Case History, *Mini-TQ* Mini Tinnitus Questionnaire, *TFI* Tinnitus Functional Index, *THI* Tinnitus Handicap Inventory, *WhoQol-BREF* World Health Organization Quality of Life – abbreviated, *BFI-2* Big Five Inventory-2, *CGI-I* Clinical Global Impression Scale – Improvement, *GUF* Questionnaire on Hypersensitivity to Sound, *PHQ-D* Patient Health Questionnaire for Depression, *SOISES* Social Isolation Electronic Survey, *ATAQ* Attitudes Towards Amplification Questionnaire, *FTQ* Fear of Tinnitus Questionnaire, *MoCA* Montreal Cognitive Assessment, *ABR* auditory brainstem response, *AMLR* auditory middle latency response

Screening and Baseline measurements as well as treatment start can be performed on the same day. In this case, all measurements are only performed once. The baseline should be maximum 4 weeks before the treatment start; otherwise, baseline measures should be repeated (without ESIT-SQ, TSCHQ, BFI-2, ATAQ, electrophysiological measurements)

^a^Declaration of consent (ICF) can be digital for the pre-screening

^b^Only for participants who were allocated to a single or combinational treatment with HA

^c^Blood samples can be taken at any time point before treatment start

### Intervention

#### Treatment conditions

The main objective of the UNITI RCT is to investigate the effects of four different interventions (SC, ST, HA, CBT) and the combinations of these interventions (CBT and HA, CBT and SC, CBT and ST, HA and SC, HA and ST, SC and ST). Internal standard operation procedures were developed, and workshop training was conducted to ensure harmonization among the participating clinical sites with regard to the procedure, technical equipment, and training of the research staff. A full description of each of the four treatments is available in the study protocol [1].

### Randomization and blinding

**Fig. 1:**
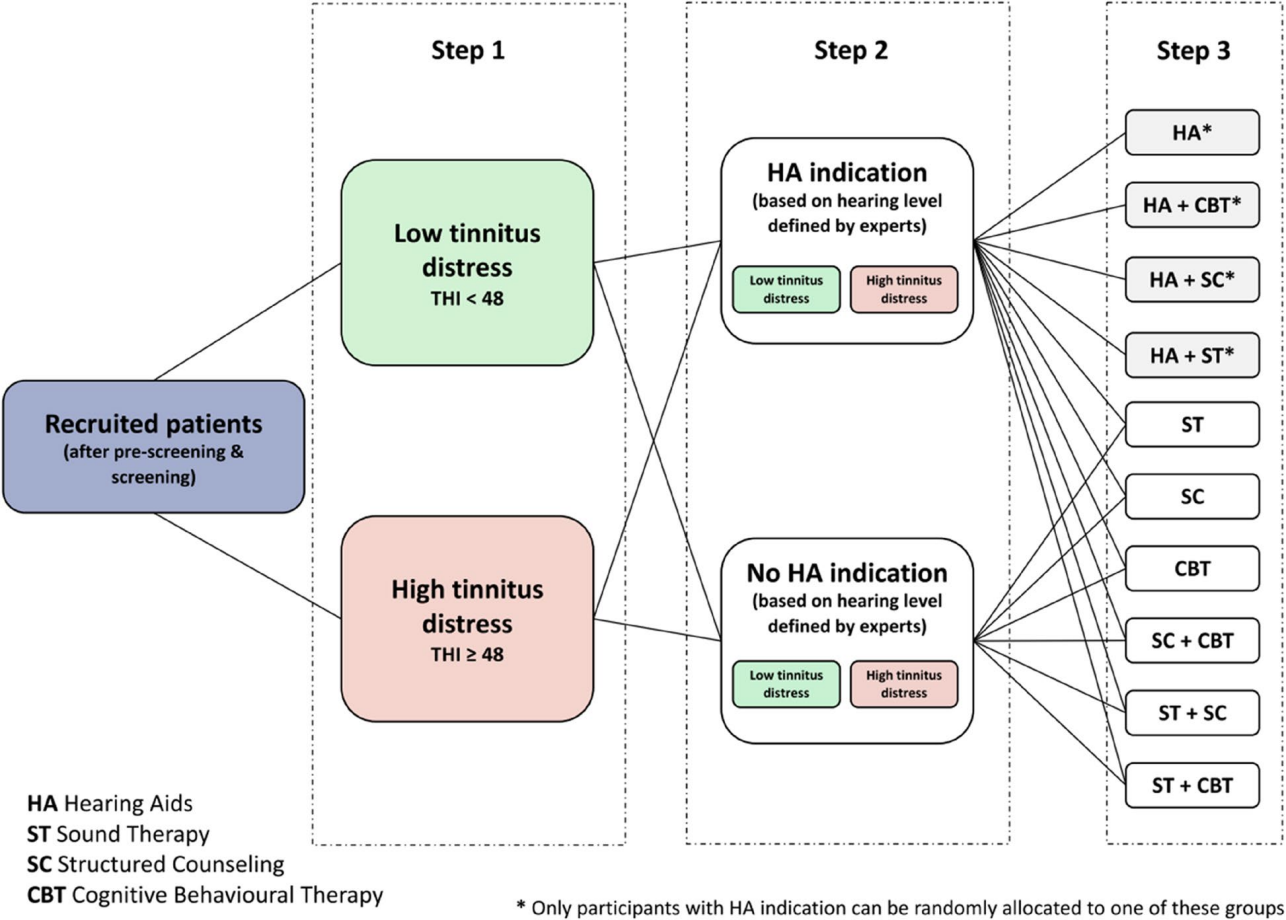
Randomization scheme as shown in the study protocol. Figure reproduced from [1] (CC BY 4.0)

Eligible participants are randomly allocated to one of ten treatment arms of single or combinational treatments (see Fig. 1). In the first step, patients are stratified into two groups according to the severity of their tinnitus distress as measured by the THI. Participants with a THI score greater or equal to 48 are allocated to a “high distress” group, whereas participants with a THI score smaller than 48 are allocated to a “low distress” group. This stratification is performed to capture the tinnitus disorder subtype, which is marked by high tinnitus-related distress [17]. In the second step, the two subgroups of low and high tinnitus distress are further stratified based on their degree of hearing loss into a subgroup with and without hearing aid indication. This results in four stratification groups, namely, HA indication and low tinnitus distress, HA indication and high tinnitus distress, no HA indication and low tinnitus distress, and no HA indication and high tinnitus distress (cf. Fig. 1). An equal ratio of 25 patients per group per clinical site is intended, resulting in a total number of 100 patients per site. Subsequently, in each center, patients are assigned to one of the ten treatment arms according to predefined randomization tables to have appropriate ratios for the planned primary analysis/contrasts (e.g., single vs. combinatory treatment).

Table 5 shows the expected allocation of patients to each of the ten treatment arms considering the proportional ratios of the planned analysis.

**Table 5:** Expected randomization per center and per treatment

	**Randomized allocation of patients in Athens**	**Randomized allocation of patients in Berlin**	**Randomized allocation of patients in Granada**	**Randomized allocation of patients in Leuven**	**Randomized allocation of patients in Regensburg**	**Total**
HA	12	12	12	12	12	60
ST	12	12	12	12	12	60
SC	12	12	12	12	12	60
CBT	12	12	12	12	12	60
HA + CBT	4	4	4	4	4	20
HA + SC	4	4	4	4	4	20
HA + ST	6	6	6	6	6	30
SC + CBT	12	12	12	12	12	60
ST + SC	14	14	14	14	14	70
ST + CBT	12	12	12	12	12	60
Total	100	100	100	100	100	500

The randomization of patients takes place at each clinical site and is monitored centrally. A specific interactive web response system (IWRS) is used to support each clinical site with the randomization of their patients. This facilitates the management of many patients from different sites located in several countries and the monitoring of the multicentric study with a complex design. The distribution across the four strata is centrally monitored during the randomization process. If a recruited and eligible participant quits the RCT participation before randomization, this participant is considered a screening failure. In case an eligible participant is already randomized to a treatment group and quits study participation, this patient is considered a dropout.

The local clinical staff will enter clinical data into a central tinnitus database [6]. Patient-specific data as well as treatment types will be stored with specific pseudo-anonymized codes. The data analysis team (see section timing of analysis) will only have access to the blinded treatment codes stored in the database and will therefore be blinded to the type of treatment participants received. The statistical analysis team will have the treatment codes unblinded only after the analysis is completed by the project coordinators (SSch and WS).

### General principles of statistical analysis

A two-sided *p*-value of < 0.05 will be considered statistically significant, and parameter estimates will be presented with two-sided 95% confidence intervals.

### Sample size calculation

A sample size of 500 participants has been calculated based on conservative estimates of the effect size from previous clinical trials delivering CBT, SC, and ST, with the aim to achieve enough statistical power to address objective 1; see the study protocol [1]. Each of the five centers will recruit 100 patients. An equal ratio between the four strata (HA yes, THI ≥ 48; HA no, THI ≥ 48; HA yes, THI < 48; HA no, THI < 48) is intended for each study site.

### Timing of analysis

An initial data exploration is conducted during data collection to ensure the integrity (i.e., the overall completeness and accuracy) of the data stored in the database. No interim analyses are planned. Data preparation, such as data cleaning (e.g., standardizing variable names, encoding categorical variables as factors) and munging will take place for each center after the final visit of the last patient is recorded, as well as plausibility checks. Exploratory data analysis with graphical methods (e.g., histograms, bar-plots, scatterplots, graphical exploration of missing values) will also be conducted for each center after the final visit of the last patient is recorded. The initial and exploratory data analysis, as well as the analysis of the main results, will be carried out by the statistical analysis team [JS, SG, CJ, UN, MSp, ME, NW, LB] with the pseudo-anonymized treatment code, and therefore, treatment blindness will be preserved. The main RCT analyses will include data from patients who have finished their treatment by 19 December 2022. After that date, the data cleaning process will begin. Secondary outcome analysis is planned to occur when the 48-week follow-up period has been reached for participants included in the primary outcome analysis.

### Statistical software

All preprocessing and statistical analysis will be conducted in R. Data wrangling will be done with the “tidyverse” packages [18].

### Datasets to be analyzed

The intention-to-treat (ITT) population includes all participants randomized regardless of compliance with the study protocol. Unless otherwise specified, the main analyses will be conducted on an intention-to-treat basis.

As a sensitivity analysis, the main analysis will be repeated in the per-protocol population. The per-protocol analysis will be conducted to detect potential effects of non-compliance and will include all subjects who met the requirements for treatment compliance (see Table 6).

**Table 6:** Definitions of non-compliance with treatment protocols

Treatment	Definition of non-compliance with treatment protocol
CBT	1) Missing the first and second CBT session 2) Participating in less than 6 of the 12 CBT sessions
ST	1) Not having played at least once each of the four stimuli categories
SC	1) Not having completed the first six chapters of the SC
HA	1) Having used HA for less than 4 h per day, on average, according to data logging

*CBT* cognitive behavior therapy, *ST* sound therapy, *SC* structured counseling, *HA* hearing aid

### Subject disposition

The flow of participants through the clinical trial stages will be shown with a diagram following the guidelines of the Consolidated Standards Of Reporting Trials (CONSORT) [19]. This will include the number of participants who were screened, excluded, randomized, dropped out before treatment start (reported per treatment arm), began the intended treatment, dropped out during treatment (reported per treatment arm), completed treatment, and were analyzed for the main objective (reported per treatment arm). Additionally, protocol deviations will be presented alongside reasons.

### Participant characteristics

Baseline participant characteristics will be presented descriptively in a standardized manner as shown in Tables 7 and 8. Participants will be described based on age, sex, education attainment (ESIT-SQ), PHQ-9 scores, THI scores, TFI scores, Mini-TQ scores, WHOQoL-Bref scores, hearing loss (audiometry), and clinical tinnitus characteristics (ESIT-SQ). Descriptive analysis will consist of mean scores followed by standard deviations for continuous variables and frequencies followed by percentages for discrete variables. Descriptive analysis will be available for baseline, interim (6 weeks after treatment start), final visits (12 weeks after treatment start), and follow-up 1 (36 weeks after treatment start).

**Table 7:** Baseline characteristics stratified based on center

Sample (*N* = , %)	Athens	Berlin	Granada	Leuven	Regensburg
**Age**					
Mean (SD)					
Missing (%)					
**Sex**					
Female					
Male					
Missing (%)					
**Education attainment (ESIT-SQ A5)**					
No school					
Primary (elementary school)					
Lower secondary (middle school)					
Upper secondary (high school)					
University or higher degree					
Missing (%)					
**PHQ-9 score**					
Mean (SD)					
Missing (%)					
**THI score**					
Mean (SD)					
Missing (%)					
**TFI score**					
Mean (SD)					
Missing (%)					
**Mini-TQ score**					
Mean (SD)					
Missing (%)					
**Physical health (WHOQOL)**					
Mean (SD)					
Missing					
**Psychological health (WHOQOL)**					
Mean (SD)					
Missing (%)					
**Social factors (WHOQOL)**					
Mean (SD)					
Missing (%)					
**Environment (WHOQOL)**					
Mean (SD)					
Missing (%)					
**Hearing loss**					
None					
Mild					
Moderate					
Severe					
Missing (%)					
**Tinnitus presentation (ESIT-SQ B2)**					
Constant					
Intermittent					
Missing (%)					
**Tinnitus duration (ESIT-SQ B3)**					
Mean (SD)					
Missing (%)

**Table 8:** Baseline characteristics stratified based on treatment received

Sample (*N* = , %)	CBT	HA	SC	ST	CBT + HA	CBT + SC	CBT + ST	HA + SC	HA + ST	SC + ST
**Age**										
Mean (SD)										
Missing (%)										
**Sex**										
Female										
Male										
Missing (%)										
**Education attainment (ESIT-SQ A5)**										
No school										
Primary (elementary school)										
Lower secondary (middle school)										
Upper secondary (high school)										
University or higher degree										
Missing (%)										
**PHQ-9 score**										
Mean (SD)										
Missing (%)										
**THI score**										
Mean (SD)										
Missing (%)										
**TFI score**										
Mean (SD)										
Missing (%)										
**Mini-TQ score**										
Mean (SD)										
Missing (%)										
**Physical health (WHOQOL)**										
Mean (SD)										
Missing										
**Psychological health (WHOQOL)**										
Mean (SD)										
Missing (%)										
**Social factors (WHOQOL)**										
Mean (SD)										
Missing (%)										
**Environment (WHOQOL)**										
Mean (SD)										
Missing (%)										
**Hearing loss**										
None										
Mild										
Moderate										
Severe										
Missing (%)										
**Tinnitus presentation (ESIT-SQ B2)**										
Constant										
Intermittent										
Missing (%)										
**Tinnitus duration (ESIT-SQ B3)**										
Mean (SD)										
Missing (%)										

### Treatment compliance/adherence and protocol deviations

Compliance with treatment protocols is defined for each treatment arm separately. For combined treatments, failing to meet the criteria for one of the arms is sufficient to identify a patient as failing to comply with the protocol. Table 6 summarizes the definitions for each of the arms. For CBT, meeting one of the two criteria presented in Table 6 is sufficient to identify a patient as non-compliant.

The number and percentage of participants compliant with treatment will be presented per treatment group. Compliance is determined by App-use log files (SC, ST), hearing aid log files (HA), and participation in treatment sessions (CBT). Acceptable compliance will be defined as ≥ 50% of the recommended intervention (participation in ≥ 6 CBT sessions including the first two, using HA four or more hours per day, on average, according to data logging, having completed at least the first 6 chapters of SC and having played at least once each of the four ST stimuli categories). Withdrawal from/compliance with the randomized intervention will be summarized using the following variables:

- Number of treatment discontinuations;
- Number of patients who decided to continue with study visits even though they canceled their treatment;
- Discontinuation reasons (where available);
- Compliance with the intervention (in percent), as described in Table 6;

All cases of protocol deviations will lead to an exclusion of the respective participant from the per protocol analysis. A list of deviations will be presented in a table including the treatment arm and details of the deviation. Protocol deviations are defined as any deviations from the study protocol [1], non-compliance with inclusion/exclusion criteria as checked during the standard visits (interim and end of treatment visits), non-compliance with treatment protocols, or errors in study conduct.

### Concomitant therapies

Type and frequency of concomitant medication and treatment will be categorized and presented descriptively.

### Main analysis

Mixed-effect models will be fitted to address the main objectives of the UNITI-RCT by considering the THI as response variable and including the corresponding objective, time point (baseline, interim visit, final visit and follow-up 1), and objective-by-time interaction as fixed effects. The coding of the objectives is described in Table 9. The mixed-effect models will be computed using the R packages “lme4” [20] and “lmerTest” [21]. There will be a separate model for each objective. Center and subject ID are included as random intercepts to account for the nested data structure. The model equation for the unadjusted model will look as follows:

$$\mathrm{lmer}(\mathrm{THI}\sim\mathrm{time\;point}\ast\mathrm{objective}+(1\;\vert\;\mathrm{center}/\mathrm{subject}))$$

**Table 9:** Objectives coding

	Number of factor levels	Description of factor levels	Description of comparison
Objective 1	2	**Single** (CBT, HA, SC, ST), **Combination** (CBT **+** HA, CBT **+** SC, CBT **+** ST, HA **+** SC, HA **+** ST, ST **+** SC)	All single vs. all combined treatments
Objective 2	10	**CBT**, **HA**, **SC**, **ST**, **CBT + HA**, **CBT + SC**, **CBT + ST**, **HA + SC**, **HA + ST**, **ST + SC**	Each treatment against each other
Objective 3 (CBT)	2	**Single CBT** (CBT), **Combination CBT** (CBT **+** HA, CBT **+** SC, CBT **+** ST)	Single CBT vs. combined treatments with CBT
Objective 3 (HA)	2	**Single HA** (HA), **Combination HA** (HA **+** CBT, HA** +** SC, HA **+** ST)	Single HA vs. combined treatments with HA
Objective 3 (SC)	2	**Single SC** (SC), **Combination SC** (SC **+** CBT, SC **+** HA, SC **+** ST)	Single SC vs. combined treatments with SC
Objective 3 (ST)	2	**Single ST** (ST), **Combination ST** (ST **+** CBT, ST **+** HA, ST **+** SC)	Single ST vs. combined treatments with ST
Objective 4 (CBT)	2	**CBT** (CBT, CBT **+** HA, CBT **+** SC, CBT **+** ST), **No CBT** (HA, SC, ST, HA **+** SC, HA **+** ST, ST **+** SC)	Treatments with CBT vs. treatments without CBT
Objective 4 (HA)	2	**HA** (HA, HA **+** CBT, HA **+** SC, HA **+** ST), **No HA** (CBT, SC, ST, CBT **+** SC, CBT **+** ST, ST **+** SC)	Treatments with HA vs. treatments without HA
Objective 4 (SC)	2	**SC** (SC, SC **+** CBT, SC **+** HA, SC **+** ST), **No SC** (CBT, HA, ST, CBT **+** HA, CBT **+** ST, HA **+** ST)	Treatments with SC vs. treatments without SC
Objective 4 (ST)	2	**ST** (ST, ST **+** CBT, ST **+** HA, ST **+** SC), **No ST** (CBT, HA, SC, CBT **+** HA, CBT **+** SC, HA **+** SC)	Treatments with ST vs. treatments without ST
Objective 5	3	**Brain** (CBT, SC, CBT** +** SC), **Ear** (HA, ST, HA **+** ST) **Brain + Ear** (CBT **+** HA, CBT **+** ST, HA **+** SC, SC **+** ST)	Ear mediated vs. brain mediated vs. ear and brain mediated treatments

The assumptions for linear mixed-effect models will be tested using diagnostic plots using the “check_model” function of the “performance” package [22]. The check includes linearity, homogeneity of variance, multicollinearity, normality of residuals, and normality of random effects. If any violations are detected visually, appropriate transformations will be performed or appropriate non-parametric/semi-parametric statistical methods will be selected, depending on the distribution of the data and the specific type of violation. Any such changes will be reported in detail.

### Adjusted analysis

In addition to the model described above, sensitivity analysis will be conducted by adjusting the model with the following fixed effects: age, gender, educational attainment, hearing aid indication, and depression according to the PHQ-9 measured during baseline. Adjusted fixed effects estimates will be reported with their 95% confidence intervals. The model equation for the adjusted model will look as follows:

$$\mathrm{lmer}(\mathrm{THI}\sim\mathrm{time\;point}\ast\mathrm{objective}\;+\;\mathrm{age}\;+\;\mathrm{gender}\;+\;\mathrm{educational\;attainment}\;+\;\mathrm{hearing\;aid\;indication}\;+\;\mathrm{PHQ9\;baseline}\;+\;(1\;\vert\;\mathrm{center/subject}))$$

Unadjusted and adjusted models will also be fitted for the secondary outcomes.

### Treatment of missing data

Multiple imputation techniques [23] will be deployed if data is assumed to be missing at random (MAR) [24]. The key concept of multiple imputation is to use the non-missing observed data to estimate plausible values for the missing data [23]. This method was selected due to its lower estimate bias, especially when compared to other techniques such as the last observation carried forward [25, 26]. Multiple imputation will be used to account for participants with missing outcome values as part of the ITT analysis. More precisely, we will first use visualization to check for any missing data pattern by observed data (e.g., by questionnaire, center, or treatment). If missing data is assumed to be MAR [27], the R package “mitml” will be used to impute the missing primary and secondary outcomes based on non-missing constant values (e.g., age, gender, educational attainment, hearing loss, tinnitus duration) with *n* = 50 imputed datasets and using "jomoImpute" as multilevel imputation method [28, 29]. If missing data is assumed to be NMAR (not missing at random), sensitivity analyses such as pattern mixture models will be considered to assess the potential bias caused by data NMAR [28]. According to Rubin’s rules, models will be applied to each imputed dataset and estimates will be pooled into an overall estimate with the corresponding confidence interval [30].

### Analysis of safety outcomes

Between-group analysis of safety outcomes will be presented descriptively, as outlined in the study protocol [1].

### Adverse events (AE)

ICD-10 codes will be used for all reported adverse events. Serious adverse events as identified by Good Clinical Practice §3 are described in terms of relatedness to treatment (yes/no) and whether the adverse event was expected (yes/no) [1]. Self-reported data are used as primary sources of AE and supported by clinical reports. If the same AE is reported by self-reports and clinical reports, only the former will be presented to avoid duplications. The following medical occurrences will be considered serious adverse events:

- Death;
- Threat to life;
- Requirement for hospitalization or extension of current hospitalization;
- Persistent disability or incapacity;
- Medically relevant events (e.g., allergy).

The number of treatment-related adverse events is reported divided by their relationship to treatment (“doubtful,” “possible,” “probably,” and “certain”).

## Conclusion

The UNITI trial will be one of the world’s largest tinnitus trials and the first to compare established standard treatments performed alone or in combination. The results of the UNITI trial will provide much-needed evidence to clinicians and are likely to influence international clinical guidelines. The planned statistical analysis is detailed here to provide transparency.

## Data Availability

All investigators from UNITI-RCT have access to the study data stored in the tinnitus database [6]. Raw data (de-identified) can be provided upon request.
